# Novel bio-catalytic degradation of endocrine disrupting compounds in wastewater

**DOI:** 10.3389/fbioe.2022.996566

**Published:** 2022-10-25

**Authors:** Budeli P, Unoufin JO, Resoketswe Charlotte Moropeng, MNB Momba

**Affiliations:** ^1^ Department of Environmental, Water and Earth Sciences, Tshwane University of Technology, Arcadia Campus, Pretoria, South Africa; ^2^ Department of Chemical Engineering, University of Pretoria, Pretoria, South Africa; ^3^ Department of Biomedical Sciences, Tshwane University of Technology, Arcadia Campus, Pretoria, South Africa

**Keywords:** Endocrine disrupting compounds, estrogens, bacteria, bio catalyst degradation, response surface methodology

## Abstract

Against the backdrop of towering ecological health implications of estrogen pollution and the inefficacies associated with cost-intensive treatment techniques, this study recorded the earliest attempt of developing an inexpensive bacterial laccase-based biocatalysts for biodegradation of EDCs (Endocrine disrupting compounds), particularly estrogens. First, a central composite design was used to investigate the interactive effects of pH (6.0–8.0), inoculum size (100–500 U/mL), and copper (Cu) (25–75 mg/L) on laccase activity and estrogen degradation respectively. Thereafter, biocatalysts was synthesized comprising laccase and glass beads or silver impregnated clay granules (SICG), which was further used to treat estrogen infused aquatic matrices under different reaction conditions. Maximum laccase activities and estrogen removal for the two tested laccases were 620 U/mL (85.8–92.9%) and 689.8 U/mL (86.8–94.6%) for *Lysinibacillus sp.* BP1 and *Lysinibacillus sp.* BP2, respectively, within 72 h, under conditions of optimal inoculum size and/or Cu concentration. Apart from a higher estrogen removal rate compared to free laccased, the biocatalysts were more resistant to temperature, pH and other environmental perturbations, and had enhanced storage ability and reusability. In comparison to clay, beads had a higher potential for recyclability and were more stable under certain experimental factors such as pH, reuse, and temperature, as well as storage conditions. Immobilized enzymes were able to remove 100% of E2, as well as over 90% of E1 and EE2, in 24 h, indicating that they could be scaled up to benchtop bioreactor levels.

## Introduction

For the elimination of EDCs, conventional methods such as adsorption, electrochemical oxidation, improved chemical oxidation, membrane filtration, and photocatalysis have been developed. However, subsequent research have revealed that these approaches have numerous downsides, including a significant environmental effect, operational difficulties, and costs (Belhaj et al., 2015; Ben et al., 2017). Although biological treatment methods are hypothetically considered the best option due to their cost and environmental friendliness, they are significantly overridden by expensive physico-chemical methods for various real-life environmental applications, including the removal of Endocrine disrupting compounds (EDCs) from contaminated sites (Budeli et al., 2020). Nevertheless, phenol oxidases, such as laccases and peroxidases, are recently receiving increased attention due to their potential to mitigate EDCs from aqueous solutions (Morsi et al., 2020; Shakerian et al., 2020). While enzymes have demonstrated their ability to detoxify EDCs in a laboratory setting, their use in real-life continuous systems such as wastewater treatment plants faces challenges such as sensitivity to natural environmental conditions, non-reusability, structure sensitivity, and sensitivity to plant operational parameters (pH, temperature, retention time, among others) (Falade et al., 2018). The majority of these bottlenecks can be circumvented by formulating biotechnologically relevant enzymes into environmentally robust composites. Thus, new research advances in this field primarily focus on optimizing and increasing enzyme stability to withstand environmental disturbances that invariably obstruct the operation of enzyme-based treatment technologies. (Daronch et al., 2020).

The type of enzyme, co-constituent and synthesis process all influence the final characteristics of biocatalysts. Adsorption, covalent binding, encapsulation, and cross-linking are the commonly applied techniques for immobilizing biotechnologically relevant oxidoreductases (Aricov et al., 2020; Aggarwal et al., 2021). There is a growing body of literature on enzyme immobilization strategies and their positive effects on enzyme catalytic activity; however, most immobilization supports are expensive, limiting their adoption, particularly in developing countries (Mohamad et al., 2015; Reis et al., 2019; Wahab et al., 2020). Therefore, using of a highly porous material with a large surface area, such as clay or alginate beads, may aid in pollutant removal *via* simultaneous adsorption and biocatalytic conversion (Daâssi et al., 2014; Falade et al., 2018; Budeli et al., 2020). Adding 1 g of laccase from *Trametes hirsuta* EDN 082 immobilized on light expanded clay aggregate to 100 ml of wastewater solution resulted in complete decolorization efficiency within 1 h (Yanto et al., 2021). Furthermore, the quantity and source of enzyme in conjunction with the use of functional materials with specified qualities increases the immobilized enzymes’ operability (Sharma et al., 2018; Moreno et al., 2020; Shakerian et al., 2020).

Previous research on xenobiotic detoxification of EDCs in wastewater using enzyme-based treatment methods focused primarily on oxidoreductases isolated from fungi and algae, despite the fact that bacteria also possess these catalytic enzymes (Macellaro et al., 2014; Falade et al., 2018; Budeli et al., 2020; Kasonga et al., 2021; Kumari et al., 2021). The paucity of references in the literature relating to application of bacterial laccase-based composite as alternative green treatment highlights the need to consider this aspect as a future research hotspot. Against this background, the current study aimed at developing novel biocatalysts for degradation of EDCs, focusing specifically on estrogen (E1, E2 and EE2). The specific objectives of the current study were to (1) evaluate EDC degrading ability of bacterial laccase as free agents using Response surface methodology (RSM) (2) optimize degradation performance through interaction of process variables such as pH, enzyme inoculum size and Cu as a co-factor (3) biocatalyst development through immobilization of identified degradative laccases on solid supports [Silver impregnated clay granules (SICG) and glass beads] (4) to assess the ability of resulting biocatalysts to degrade EDCs in a continuous fed batch reactor under harsh conditions, pH and temperature variation tolerance, storage conditions and reusability.

## Materials and methods

### Chemicals and solvents

All reagents, cartridges, and filter papers were purchased from Sigma-Aldrich (Sigma-Aldrich, St. Louis, MO, United States), as were the compound standards (>98%) for E1, E2, and EE2. Bisphenol A-d16 was used as an internal standard, as well as HPLC grade methanol, ethanol, and acetone, formic acid, and hydrochloric acid (99% purity). Sigma-Aldrich also provided ABTS (2,2′-Azinobis [3-ethylbenzothiazoline-6-sulfonic acid]-diammonium salt), Tri-chloro acetic acid (TCA), and glass beads. SICG granules were synthesized at CSIR SA (Material science unit) as described by Budeli et al. (2021). The SICG employed in this work was undertaken in partnership with CSIR CERMALAB material and testing laboratory for the manufacture of the silver-impregnated granules. Briefly, natural clay was mixed with sawdust and paper fibre infused with silver nitrate (AgNO_3_), followed by moulding into microscopic granules. The mixture was fired at 887 °C for 8–9 h, then incubated at 90 °C for 48 h to allow the granules to dry out. Finally, the dried clay granules were rinsed with synthetic water containing 0.9 percent w/v salt water and re-dried at room temperature.

### Innoculum and laccase activity

The screening, isolation and characterisation of strains used in this study has been described elsewhere (Budeli et al. unpublished). The choice isolates, based on the aforementioned processes, were identified as *Lysinibacillus* sp. BP1 and *Lysinibacillus* sp. BP2, respectively. Measurement of laccase activity was according to an previous study (Unuofin et al., 2019b). Briefly, 100 ml of crude laccase was incubated in 2 mM ABTS in pH 6 potassium phosphate buffer, which was allowed to react for 10 min and stopped using 40 ml 20% TCA. Thereafter, the reaction was read spectrophotometrically (Shimadzu UV-2450) at 420 nm and laccase activity (international unit: IU) was interpreted as the amount of enzyme required to catalyse 1 µmol ABTS oxidation per minute under standard assay conditions. Controls comprised crude extracts from bacterial cells grown under the same conditions, but without estrogens. However, twenty (20 mg) of glass beads and SICG were incubated in 1 ml of 2 mM ABTS in 100 mM sodium citrate buffer to determine immobilized enzyme activity under optimal conditions.

### Estrogen degradation and optimization of parameters using RSM

Based on optimal parameters observed from a one-variable-at-a-time (OVAT) approach in a previous study (Budeli et al. unpublished), estrogen degradation was further optimized by computing the interactive effects of estrogen concentration (100–500 ng/L), pH (6.0–8.0), inoculum (100–500 µL) and copper (25–75 mg/L) in a central composite design (CCD), where responses measured were laccase activity (R1), E1 degradation (R2), E2 degradation (R3) and EE2 degradation (R4). Design expert software v13 trial version (Minneapolis, United States) was used to evaluate the design in terms of single, interaction, and quadratic effects. Each factor was varied at three levels: 1, 0, and +1, corresponding to low, medium, and high values. The design consisted of 30 duplicated runs to optimize the levels of the selected variables. The experiment was run for 72 h; thereafter, aliquots of pure estrogens and their degraded residual concentrations were collected and analysed using LCMS-MS.

### Calculation of the removal efficiency of target EDCs

The degradation efficiencies of the laccases were calculated using the equation presented below.

$$\mathrm{Removal}\;\mathrm{efficiency}\;\left(\%\right)=\frac{C\;inf-C\;eff}{C\;inf}x\;100$$

Where C_inf_ is the initial concentration and C_eff_ is the final concentration (Kibambe et al., 2020).

### Immobilization of laccases on beads and SICG for biocatalyc development

SICG was crushed with a crusher and pestle before to immobilization to obtain smaller particles. Glass beads ranging between 0.4–0.6 mm in diameter (Zymobics, South Africa) were pre-treated with 1.2 M HNO_3_ at 60 C for 4 h before being thoroughly washed with water and dried at 60°C. A 10 ml solution of glutaraldehyde (10%) was added to the prepared clay and glass beads and incubated at room temperature for about 5 h. The particles were washed with deionized water to remove unbound laccases and excess glutaraldehyde and thereafter airdried. Immobilization yield was calculated by dividing the laccase activity measured in the biocatalysts by the total activity present in the liquid solution at the start of the immobilization process. This procedure resulted in yields of over 76 and 84% for glass beads and SICG, respectively.

### Storage stability of free and immobilized activity of laccases against temperature and pH

The storage stability of free and immobilized laccases was investigated by measuring enzyme activity during storage at 4 or 25°C for 30 cycles. The residual enzyme activities of free and immobilized laccases were measured after only 60 min of incubation with 0.1 M phosphate buffer (pH 6) at temperatures ranging from 10 to 100°C. Laccase activity was measured at room temperature after incubation, as previously described. The effect of pH on enzyme activity was studied at 25°C in the pH range of 2–10.

### Degradation and reusability of the designed biocatalysts

Degradation ability of the designed biocatalysts was evaluated using a laboratory scale continuous fed-batch reactor (250 ml filtration collection flask). About 200 mg of both biocatalysts corresponding to 500 U/mL were added to the reactors separately. Thereafter, reactors were separately fed with a 100 ml solution containing mixture of estrogens (500 ng/L) in deionized water under optimised conditions. The aliquots were withdrawn using a 1,000 ml micropipette at 24 h intervals to evaluate enzymatic activities and estrogen degradation. An immobilized enzyme and a number of beads corresponding to the appropriate enzymatic activity were used to breakdown the EDCs combination. Repeated measurements (30 cycles) of the activities of designed biocatalysts at room temperature were used to assess the recyclability of the resulting biocatalysts. The reactors were emptied using a filtration vacuum pump after each cycle and washed three times (30 min interval) with deionized water before the next feed. The reactors were placed in a shaking incubated at an agitation speed of 100 rpm to create perturbation. The removal efficiencies of developed biocatalysts were calculated using the aforementioned equation presented below.

$$\mathrm{Removal}\;\mathrm{efficiency}\;\left(\%\right)=\frac{C\;inf-C\;eff}{C\;inf}x\;100$$

### Statistical analysis

Statistical analyses of data synthesized in the current study were performed using Design expert software v13 trial version (Minneapolis, United States) and microsoft Excel 2010.

## Results

### Free crude laccase production and degradation of EDCs

**FIGURE 1:**
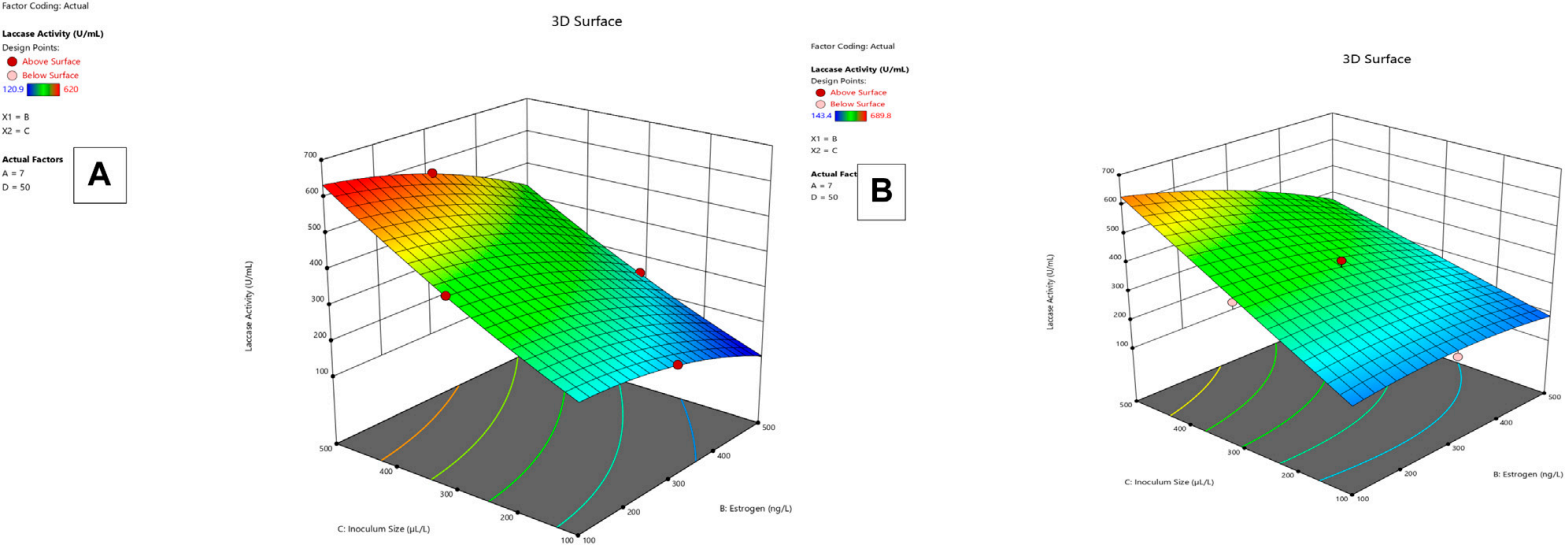
R1 and R2 response 3D mesh diagram for both *Lysinibacillus.* BP1 **(A)** and *Lysinibacillus.* BP2 **(B)**.

**FIGURE 2:**
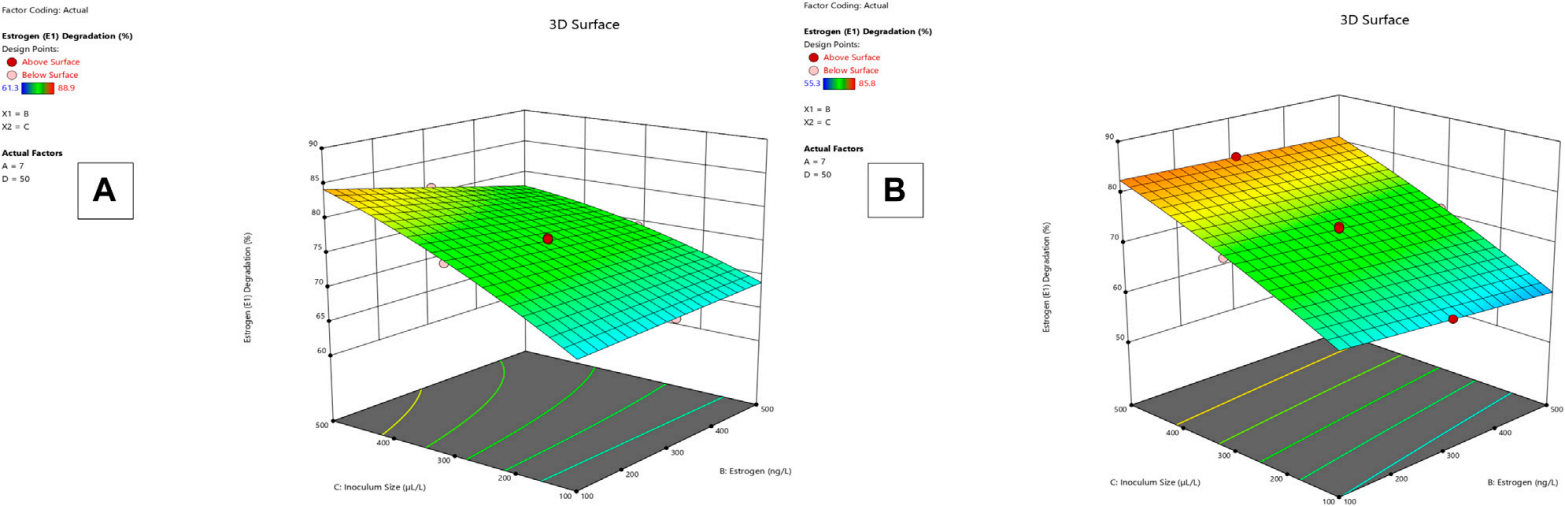
E1 degradation 3D mesh diagram for *Lysinibacillus.* BP1 **(A)** and *Lysinibacillus.* BP2 **(B)**.

**FIGURE 3:**
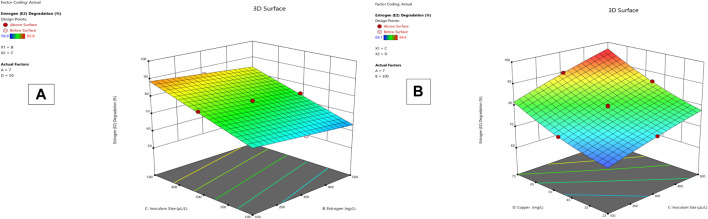
E2 degradation 3D mesh diagram for *Lysinibacillus.* BP1 **(A)** and *Lysinibacillus.* BP2 **(B)**.

**FIGURE 4:**
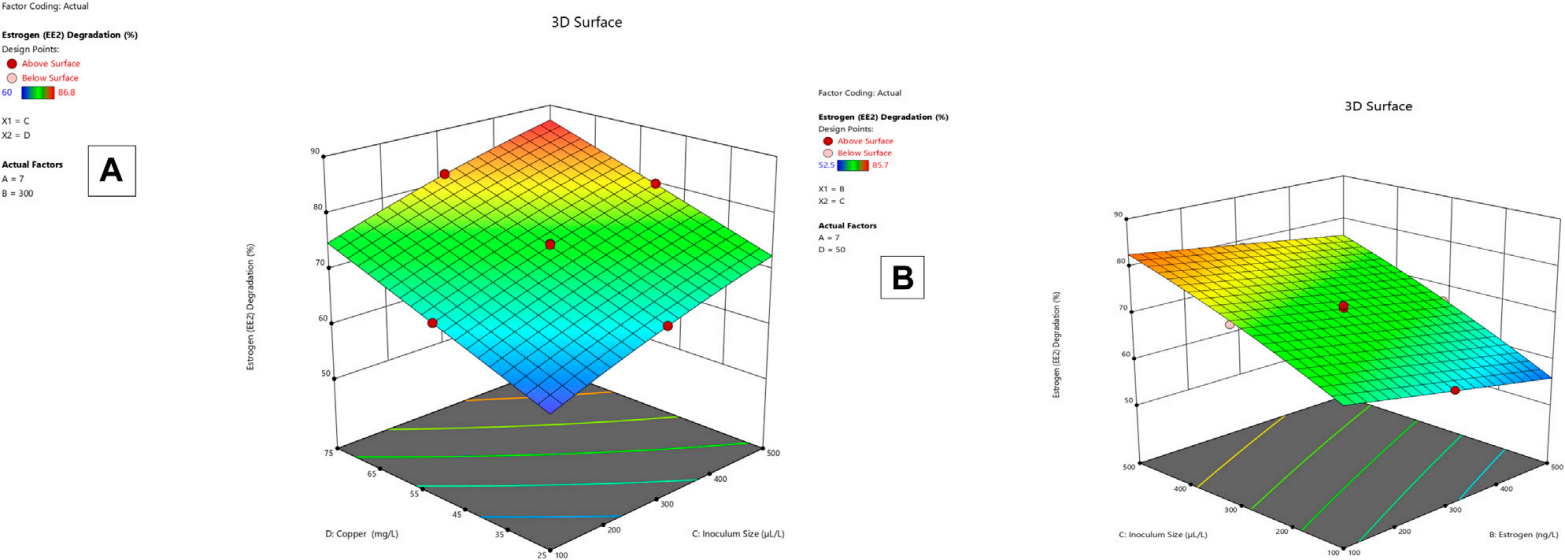
EE2 degradation 3D mesh diagram for *Lysinibacillus.* BP1 **(A)** and *Lysinibacillus.* BP2 **(B)**.

*Lysinibacillus.* BP1 elicited maximum enzymatic activity (689.8 U/mL) and degradation (86.8–94.6%) at pH 6.0, 100 ng/L estrogen concentration, 500 µL and Cu concentration of 75 mg/L. (Table 1). Conversely, *Lysinibacillus.* BP2 demonstrated optimal enzymatic activities of (620 U/mL) and degradation (85.7–92.9%) under the central values of all the factors evaluated (Table 2). Among selected single factors, B (inoculum dosage) and Cu were found to be significant. The significant interactions during enzymatic degradation of estrogens in both R1 and R2 are presented in a 3D mesh (Figures 1–4). From the mesh diagrams, it was observed that an inverse relationship between inoculum size and estrogen concentration favoured enhanced laccase production for both isolates, at constant pH and copper concentration (Figure 1). An advancement in inoculum size and a corresponding decreases in estrogen concentration improved laccase activity. A similar trend was observed during E1 degradation (Figure 2). In addition to this trend, we also observed that a parallel relationship between copper concentration and inoculum size general enhanced degradation of all estrogens assessed; this is exemplifiedin Figure 3B, 4A.

**TABLE 1:** CCD matrix showing actual and predicted response for *Lysinibacillus* sp. BP1 estrogen degradation.

Run	A:pH	B:Estrogen ng/L	C: Inoculum µL/L	D:Copper mg/L	Enzymatic activities U/mL		E1 degradation (%)		E2 degradation (%)		EE2 degradation (%)	
					Actual	Predicted	Actual	Predicted	Actual	Predicted	Actual	Predicted
1	7	300	100	50	213.9	211.94	62.3	62.23	66.4	66.65	60.9	60.77
2	8	500	100	75	170.3	171.47	60.9	60.76	64.4	64.19	58.5	58.93
3	8	500	100	25	120.9	121.17	55.3	54.90	58.9	59.01	52.5	52.58
4	8	300	300	50	368.1	365.91	72.6	73.03	75.7	75.62	70.7	70.92
5	7	300	300	50	368.3	370.90	73.0	72.98	75.8	76.50	70.9	71.11
6	8	100	500	75	619.3	619.72	84.9	84.20	91.2	90.84	84.7	84.94
7	7	300	300	50	368.9	370.90	73.2	72.98	75.9	76.50	70.9	71.11
8	6	100	500	75	620.0	620.07	85.8	86.63	92.9	93.29	85.7	85.24
9	6	100	100	75	254.1	255.27	71.6	71.86	74.8	75.06	67.2	67.48
10	6	300	300	50	368.0	366.27	72.5	72.93	75.7	75.62	70.5	71.30
11	6	500	100	75	172.5	172.50	61.1	61.16	65.7	69.94	59.7	59.38
12	7	100	300	50	427.3	425.34	73.8	74.28	79.7	79.10	74.3	75.09
13	7	500	300	50	253.1	251.14	71.2	71.68	74.5	73.90	66.7	67.13
14	7	300	300	25	352.2	350.24	71.9	71.71	75.7	75.62	70.4	70.31
15	8	500	500	25	446.4	446.77	73.9	73.75	80.1	80.01	75.6	75.28
16	8	500	500	75	492.3	492.57	76.8	77.11	80.6	80.84	76.7	76.63
17	8	100	500	25	588.9	589.02	83.1	83.11	88.9	89.16	80.1	80.09
18	6	100	500	25	589.0	589.37	83.6	83.51	89.3	89.06	80.4	80.24
19	7	300	300	50	369.0	370.90	73.5	72.98	76.2	76.50	71.7	71.11
20	7	300	300	75	417.9	415.94	73.6	74.26	79.4	79.32	72.2	71.19
21	8	100	100	25	218.8	219.07	63.8	65.30	68.6	68.99	63.5	63.48
22	7	300	300	50	369.3	370.90	73.6	72.98	77.8	76.50	71.9	71.11
23	7	300	300	50	369.1	370.90	73.6	72.98	76.4	76.50	71.9	71.11
24	7	300	500	50	569.6	567.64	81.9	81.83	86.1	86.35	78.8	79.42
25	8	100	100	75	255.6	254.92	71.8	71.46	75.4	75.16	67.6	67.33
26	6	100	100	25	219.2	219.42	69.1	67.73	71.8	71.44	63.8	63.78
27	6	500	500	25	447.0	447.12	75.9	76.18	80.3	80.41	75.7	75.88
28	6	500	500	75	492.7	492.92	77.9	77.51	82.8	82.59	77.1	77.08
29	7	300	300	50	369.0	370.90	73.3	72.98	76.0	76.50	71.2	71.11
30	6	500	100	25	121.3	121.52	56.8	57.33	59.5	59.41	53.2	53.18

**TABLE 2:** CCD matrix showing actual and predicted responses for *Lysinibacillus*. BP2 estrogen degradation.

Run	A:pH	B:Estrogen ng/L	C: Inoculum µL/L	D:Copper mg/L	Enzymatic activity U/mL		E1 degradation (%)		E2 degradation (%)		EE2 degradation (%)	
					Actual	Predicted	Actual	Predicted	Actual	Predicted	Actual	Predicted
1	7	300	100	50	201.2	223.87	68.3	68.43	72.7	72.61	67.5	67.45
2	8	500	100	75	455.6	452.45	78.1	78.08	80.6	80.64	75.6	75.56
3	8	500	100	25	192.1	192.20	65.8	66.07	68.1	68.09	60.4	60.43
4	8	300	300	50	274.7	289.96	74.8	75.29	79.1	79.01	74.0	74.04
5	7	300	300	50	399.5	377.42	76.3	75.99	79.3	79.62	74.2	74.42
6	8	100	500	25	424.7	412.25	77.2	76.97	80.4	80.39	74.3	74.33
7	7	300	300	50	399.6	377.42	76.3	75.99	79.4	79.62	74.6	74.42
8	6	100	500	25	400.0	408.63	76.4	76.76	80.1	80.14	74.8	74.78
9	6	100	100	75	224.8	221.43	71.0	70.98	74.2	74.24	69.7	69.68
10	6	300	300	50	399.2	414.46	76.2	76.69	79.3	79.21	74.2	74.06
11	6	500	100	75	449.5	448.83	77.3	77.15	80.5	80.49	75.6	75.65
12	7	100	300	50	400.6	415.86	77.0	77.34	80.3	80.21	76.3	76.25
13	7	500	300	50	269.2	284.46	74.3	74.64	77.3	77.21	71.9	71.85
14	7	300	300	25	214.8	230.06	69.2	69.28	73.0	72.91	67.0	66.95
15	8	500	500	25	271.1	271.75	74.5	74.58	78.2	78.24	72.2	72.16
16	8	500	500	75	639.6	639.65	87.3	87.45	92.6	92.59	83.9	83.93
17	8	100	500	75	689.8	689.40	88.9	88.61	94.6	94.64	86.8	86.76
18	6	100	500	75	689.2	685.78	88.3	88.45	94.2	94.19	86.4	86.45
19	7	300	300	50	372.5	377.42	76.1	75.99	79.3	79.62	74.2	74.42
20	7	300	300	75	612.6	627.86	84.2	84.28	89.9	89.81	81.8	81.75
21	8	100	100	25	143.4	143.75	61.3	61.13	66.1	66.14	60.0	59.96
22	7	300	300	50	399.8	377.42	76.3	75.99	79.7	79.62	74.8	74.42
23	7	300	300	50	399.2	377.42	76.3	75.99	79.3	79.62	74.2	74.42
24	7	300	500	50	508.3	530.97	81.0	81.03	85.7	85.61	80.0	79.95
25	8	100	100	75	225.5	225.05	71.8	71.92	74.9	74.89	69.9	69.93
26	6	100	100	25	144.3	140.13	61.7	61.7	66.5	64.49	60.3	60.35
27	6	500	500	25	272.6	268.13	74.6	74.37	78.5	78.49	72.0	72.05
28	6	500	500	75	639.9	636.03	87.3	87.28	92.8	92.84	83.9	83.88
29	7	300	300	50	400.3	377.42	76.5	75.99	80.2	79.62	74.2	74.42
30	6	500	100	25	192.5	188.58	66.8	66.63	68.7	68.74	60.4	60.38

### ANOVA for response surface reduced model

Analysis of variance (ANOVA) for the quadratic model showed F-value of 131.88 in the case of enzymatic activity response. The significance of the model was validated by the p values less than of 0.0500 which were noted to be less than 0.05 in all the cases as shown in Table 3. The adequate precision was found to be greater than 5 in both responses. The correlation coefficient values were found to be quite close to 1.0 (0.9930 and 0.9854).

**TABLE 3:** Reduced cubic ANOVA model for Lysinibacillus. BP1 (A) laccase activity

Source	Sum of	Df	Mean	F	p-value	
	Squares		Square	Value	Prob > F	
Model	6.207E+05	15	41378.91	9802.99	< 0.0001	significant
A-pH	0.5689	1	0.5869	0.1348	0.7190	
B-Estrogen	15172.82	1	15172.82	3594.56	< 0.0001	
C-Inoculum size	63261.24	1	63261.24	14987.08	< 0.0001	
D-Copper	2158.24	1	2158.24	511.31	< 0.0001	
BC	1938.20	1	1938.20	459.17	< 0.0001	
BD	2067.98	1	2067.98	489.92	< 0.0001	
CD	1,262.03	1	1,262.03	298.98	< 0.0001	
A2	59.89	1	59.89	14.19	0.0021	
B2	2763.30	1	2763.30	654.65	< 0.0001	
C2	924.73	1	924.73	219.07	< 0.0001	
D2	385.13	1	385.13	91.24	< 0.0001	
BCD	962.55	1	962.55	228.04	< 0.0001	
A2B	1,681.68	1	1,681.68	398.40	< 0.0001	
A2C	47.50	1	47.50	11.25	0.0047	
A2D	724.96	1	724.96	171.75	< 0.0001	
Residual	59.09	14	4.22			
Lack of Fit	58.52	9	6.50	56.71	0.0002	significant
Pure Error	0.5733	5	0.1147			
Cor Total	6.207E+05	29				

In this case A, B, C, D, BC, BD, CD, A^2^, B^2^, D^2^, BCD, A^2^B, A^2^D, AB^2^ are significant model terms. Based on the statistical significance, the quadratic equations can be written as:

Lysinibacillus*.* BP1 laccase activity (U/mL) = 370.896 + -0.177778 * A + -87.1 *B + 177.85 *C + 32.85 *D + -11.0062 * BC + 11.3688 * BD + -8.88125 * CD + -4.80789 * A^2^ + -32.6579 *B^2^ + 18.8921 *C^2^ + 12.1921 *D^2^ + 7.75625 * BCD + 30.7563 * A^2^B + -5.16875 * A^2^C + -20.1938 * A^2^D

Lysinibacillus. BP2 laccase activity (U/mL) = 377.418 + -62.25 * A + -65.7 *B + 153.55 *C + 198.9 *D + -58.2625 * BC + 33.7125 * BD + 37.9375 * CD + -25.2045 * A2 + -27.2545 *B2 + 51.5455 *D2 + -11.025 * BCD + 76.4 * A2B + -28.6 * A2C + -75.575 * A2D + 64.0625 * AB2

BP1 E1 degradation (%) = 72.9833 + 0.05 * A + -1.3 *B + 9.8 *C + 1.27778 *D + 0.50625 * AD + 0.58125 * BC + 0.51875 * BD + -1.21875 * CD + -0.955556 *C^2^ + 0.59375 * BCD + -3.39375 * A^2^B + -1.58125 * A^2^C + -0.75625 * AB^2^

BP2 E1 Degradation (%) = 75.9946 + -0.7 * A + -1.35 *B + 6.3 *C + 7.5 *D + 0.18125 * AD + -1.83125 * BC + 0.30625 * BD + 0.40625 * CD + -1.26774 *C2 + 0.782258 *D2 + -0.19375 * ACD + 2.29375 * A2B + -1.76875 * A2D + 0.79375 * AB2

BP1 E2 degradation (%) = 76.4968 + 5.23364e-16 * A + -2.6 *B + 9.85 *C + 1.85 *D + 0.025 * AB + 4.55267e-16 * AC + 0.15 * AD + 0.225 * BC + 0.675 * BD + -1.525 * CD + -0.880645 * A^2^ + 0.969355 *D^2^ + -0.4875 * ABD + 0.4375 * BCD + -2.4375 * A^2^B + -0.6625 * A^2^C + -0.6875 * A^2^D + -0.5625 * AB^2^

BP1 EE2 degradation (%) = 71.1083 + -0.188889 * A + -3.97778 *B + 9.32778 *C + 0.8 *D + -0.075 * AB + 2.41703e-15 * AC + 0.0375 * AD + 0.775 * BC + 1.0875 * BD + -1.7125 * CD + -1.01389 *C^2^ + 0.4625 * BCD

BP2 EE2 Degradation (%) = 74.4175 + -0.0111111 * A + -2.2 *B + 6.25 *C + 7.4 *D + 0.025 * AB + 0.0125 * AC + 0.075 * AD + -1.4 * BC + 0.6625 * BD + -0.125 * CD + -0.368421 * A^2^ + -0.368421 *B^2^ + -0.718421 *C^2^ + -0.0684211 *D^2^ + 0.0125 * ABC + -0.1 * ABD + 0.0125 * ACD + -0.725 * BCD + 2.3125 * A^2^B + 0.15 * A^2^C + -1.3125 * A^2^D

### Storage stability of free and immobilized laccase

**FIGURE 5:**
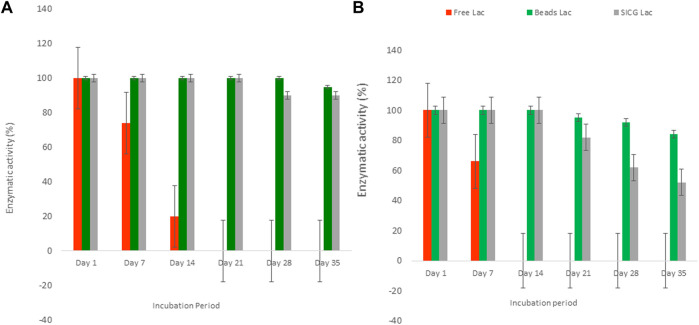
Storage stability of free and immobilized laccase at 25 **(A)** and 4°C **(B)**.

As depicted in Figure 5 below, free laccase activity showed a gradual decrease from cycle 7 which completely diminished at cycle 14 at 4 °C. A contrary trend was observed at 25°C where free laccase activities decreased from cycle 14 and only completely exhausted at cycle 21. Both immobilized beads and SICG retained their activities throughout the course of the experiment, which only started to decrease gradually from cycle 21 and 28 at 4 and 25°C, respectively. Generally, immobilised beads demonstrated higher enzymatic activities in both experimental conditions compared to SICG.

### Thermal stability of free and immobilized laccase

**FIGURE 6:**
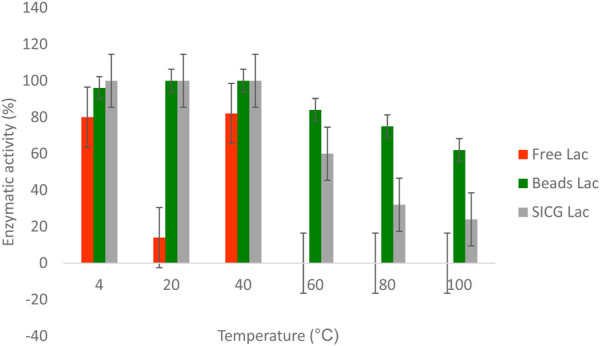
Thermal stability of laccase under different temperature levels (4–100°C).

The stability of both free and immobilised were studied and compared through incubation with buffer under varying thermal conditions (10–100°C) for 1 h. As shown in Figure 6 below, the three enzyme forms tested had different thermal resistant abilities. Free enzyme form was more sensitive to temperature change compared to immobilised forms. Interestingly, free enzyme laccase was able to retain 80% of its activity at 4°C, 100% at 20°C and 82% at 40°C. Enzymatic activities of free forms diminished completely from 60 to 100°C. Similarly, both catalysts retained 100% of their activities at 4 and 20°C. A gradual decline of activity was observed from 60°C to 100°C.

### Effect of pH on enzyme activity

**FIGURE 7:**
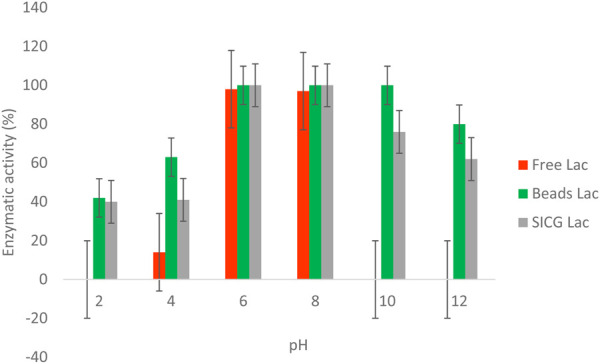
Effect of pH variation on free laccase and immobilised activities.

Enzyme activities of both free and immobilised were evaluated over a broad pH range (2–10) as shown in Figure 7. Notably, there was no enzymatic activity observed at pH 2 in free enzyme forms. The maximum activity was observed at pH 6 and 8, followed by a significant decrease from 10 to 12. Both immobilized enzyme forms demonstrated optimal activities (100%) at pH 6 and 8. Beads immobilized laccase only lost 20% of its activity at pH 12, which is significantly higher in comparison to SICG immobilized laccase that retained 62% under the same pH conditions.

### Reusability of immobilized laccase

**FIGURE 8:**
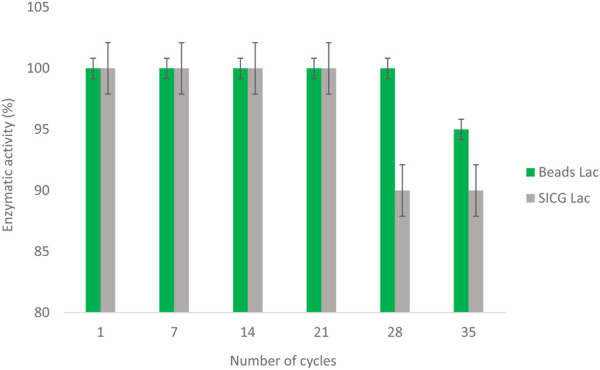
Residual activity of free and immobilized biocatalysts during 35 cycles monitoring.

As shown in Figure 8, both biocatalysts were able to retain their activity after multiple cycles. The beads immobilized laccase retained 94% of its activity after 28 cycles, while only 6% activity loss was observed at cycle 35. On the other hand, SICG immobilised laccase lost 12% of its activity after 21 cycles, which remained constant throughout until cycle 35.

### Biocatalytic degradation of EDCs in wastewater

**FIGURE 9:**
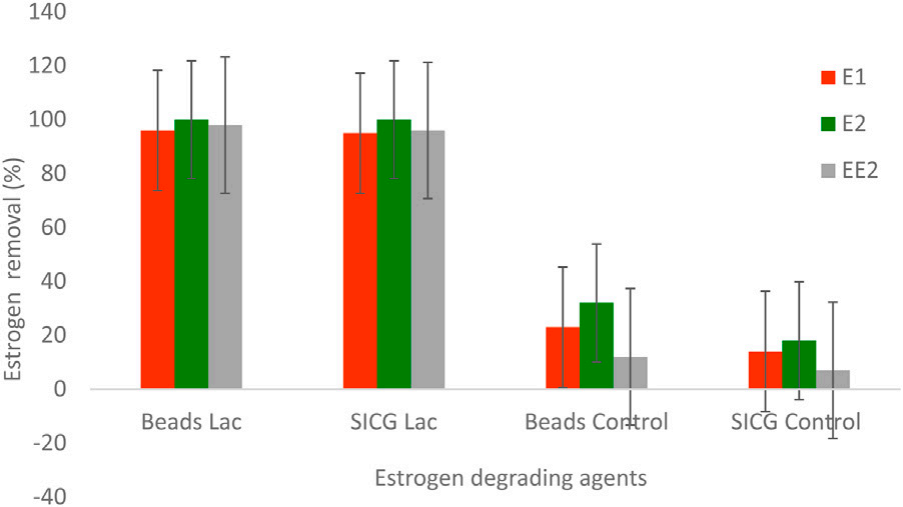
Removal of EDCs in a lab-scale continuous fed batch bioreactor using immobilized laccase.

The efficiency of the immobilized laccases in the removal of estrogen in a medium containing mixture of estrogen. Both immobilized enzymes demonstrated a 100% removal for E2 and over 90% of E1 and EE2, within 24 h. EE2 was the least removed estrogen under both circumstances, followed by E1 as shown in Figure 9.In terms of catalytic degradation of immobilization supports, beads recorded a slightly higher degradation (12–32%) compared to SICG (7–18%). Generally, beads immobilized laccase demonstrated a higher removal efficiency in comparison to SICG immobilized laccase.

## Discussion

The role of a putative bacterial laccase enzyme in EDC biodegradation is investigated in this study. This is the first known bacterial laccase enzyme that degrades estrogen, to our knowledge. Laccase is best known for catalyzing the oxidation of aromatic compounds and other classes of EDCs in lignin-biodegrading fungi (Wang et al., 2018; Singh et al., 2021). The sequences described in this study were deposited in Gen Bank data library Supplementary Table S7.2. Because of their ability to withstand high estrogen concentrations and high activity secretion of laccases known to be capable of EDC biodegradation, the two isolated isolates were chosen as potent EDC-degrading. This genus of bacteria is widely distributed in the environment and has been implicated in the degradation of micro pollutants such as aromatics, PFCs, and other chemicals (Agrawal et al., 2020; Bhatt et al., 2021). Despite the growing body of literature devoted to isolating estrogen-degrading species, there is no record in the literature implicating these isolates in EDC bioremediation. Bioremediation using whole organism processes for micropollutant removal has recently been linked to the introduction of resistant genes in wastewater (Zhang et al., 2016; Latif, 2018) as a result zymoremediation has gained more attention as a biological process alternative. The ligninolytic potential of bacteria is still largely untapped, and many novel ligninolytic enzymes may be discovered in the future. These bacterial enzymes may outperform their fungal counterparts in terms of specificity, thermostability, and mediator dependency. They may possess advantages in depolymerizing modified lignin residues, which are commonly found in waste streams from the pulping and other bio-based chemicals industries (Naz et al., 2015; Budeli et al., 2020).

Laccase is best known for catalyzing the oxidation of aromatic compounds and other classes of EDCs in lignin-biodegrading fungi (Wang et al., 2018; Singh et al., 2021). However, to our knowledge, there was no preceding information regarding estrogen degradation using bacterial laccases, this motivated our investigation of the role of putative bacterial laccase biocatalysts in EDC biodegradation. The bacterial isolates were selected based on their tolerance of high estrogen concentrations and enhanced secretion of laccases known to be capable of EDC biodegradation. Bacteria belonging to *Lysinibacillus* genus are widely distributed in the environment and have been implicated in the degradation of micro pollutants such as aromatics, PFCs, and other chemicals (Agrawal et al., 2020; Bhatt et al., 2021). Despite the growing body of literature devoted to isolating estrogen-degrading species, there is no record in the literature implicating these isolates in EDC bioremediation. Bioremediation using whole organism processes for micropollutant removal has recently been linked to the introduction of resistant genes in wastewater (Zhang et al., 2016; Latif, 2018); as a result, zymoremediation has gained more attention as an alternative process of biological treatment. The ligninolytic potential of bacteria is still largely untapped, and many novel ligninolytic enzymes may be discovered in the future. These bacterial enzymes may outperform their fungal counterparts in terms of specificity, thermostability, and mediator dependency (Naz et al., 2015; Budeli et al., 2020).

In the current study, response surface methodology-based optimization of laccase production from bacterial isolates was achieved using ABTS as a substrate. The maximum activity of the laccases assessed were 620 U/mL and 684.8 U/mL, respectively, for *L. fusiformis* and *L. macrolides*. More than 80% of the estrogen concentrations in the range of 100–500 ng/L were degraded as shown in Table 4. Maximum copper concentration (75 mg/L), estrogen concentration (100 ng/L), laccase activity (500 U/mL), and degradation (84%) and (92%) for both *L. fursiformis* and *L. macrolides*, respectively, were found at central values of all factors, namely pH 6.0, estrogen concentration (100 ng/L), activity greater than 600 U/mL, and copper concentration (75 mg/L). The current study also discovered that high estrogen concentrations have a negative impact on laccase enzymatic activity as well as estrogen degradation efficiency. In contrast, increased copper concentrations resulted in laccase induction as shown in Figure 4A–B. Because laccase is a copper-binding enzyme with four binding sites that may contribute to its oxidizing activity, copper affects laccase activity and positively contributes to the overall efficiency of biodegradation (Lin et al., 2016; Yin et al., 2019). Thus, the addition of copper during bioaugmentation in wastewater treatment may improve overall performance by inducing laccase activities in the media; however, the effect on the entire wastewater microbial community must be determined first. Some of the chemical compounds applied have been linked to the activation of antibiotic resistance genes, as well as possessing negative effects on the richness and diversity of microbial communities (Tang et al., 2016), negatively impacting the full functioning of activated sludge and other microbial-based unit processes.

**TABLE 4:** Reduced cubic ANOVA model for Lysinibacillus. BP1 (B) laccase activity.

Source	Sum of	df	Mean	F	p-value	
	Squares		Square	Value	Prob > F	
Model	7.432E+05	15	49548.94	131.88	< 0.0001	significant
A-pH	7750.12	1	7750.12	20.63	0.0005	
B-Estrogen	8632.98	1	8632.98	22.98	0.0003	
C-Inoculum size	47155.21	1	47155.21	125.51	< 0.0001	
D-Copper	79122.42	1	79122.42	210.60	< 0.0001	
BC	54312.30	1	54312.30	144.56	< 0.0001	
BD	18184.34	1	18184.34	48.40	< 0.0001	
CD	23028.06	1	23028.06	61.29	< 0.0001	
A2	1803.34	1	1803.34	4.80	0.0459	
B2	2108.63	1	2108.63	5.61	0.0327	
D2	7542.26	1	7542.26	20.08	0.0005	
BCD	1944.81	1	1944.81	5.18	0.0391	
A2B	10376.82	1	10376.82	27.62	0.0001	
A2C	1,454.15	1	1,454.15	3.87	0.0693	
A2D	10153.92	1	10153.92	27.03	0.0001	
AB2	7296.01	1	7296.01	19.42	0.0006	
Residual	5259.78	14	375.70			
Lack of Fit	4643.48	9	515.94	4.19	0.0647	not significant
Pure Error	616.29	5	123.26			
Cor Total	7.485E+05	29				

Inoculum dosage (B) and Cu concentration variation (C) had the maximum impact on laccase activity and degradation (residual estrogen concentration) thereby signifying their predominant role in estrogen degradation process. In contrast, pH (factor A) has the least significant contribution, which could be attributed to the study’s narrow pH range (pH 6–8). According to research, a large number of microbes with the ability to degrade micro pollutants are mesophilic, with maximum metabolic activities at pH levels between 6 and 8. *S. lavendulae* and *T. versicolor* laccases, on the other hand, were at their peak around pH 6 and declined slightly at pH 8 (Singh et al., 2020; Xu et al., 2020). Thus, it conceivable that a source from which the enzyme was isolated plays a crucial role in pH stability of the enzyme during biodegradation. The interaction factor BC and all the quadratic factors were also noted. The equation in terms of coded factors can be used to make predictions about the response for given levels of each factor. By default, the high levels of the factors are coded as +1 and the low levels are coded as -1. The coded equation is useful for identifying the relative impact of the factors by comparing the factor coefficients as shown in equations.

Enzymatic activities of the two isolates revealed unequivocal involvement of bacterial laccases during estrogen degradation, implying that they could serve as alternative biological sources for estrogen removal in wastewater. Studies on their antagonistic effects on wastewater microbial communities are relevant to biotechnology applications. Enzyme storage stability is pivotal in industrial applications *(Bilal et al*>, 2018). To evaluate the storage stability of laccase, we stored free and immobilized peroxidase at 4 and 25°C for 35 cycles and measured their activities. These findings indicated that immobilized laccase is far more stable in storage than free form enzyme. As shown in Figures 5A,B, beads laccase demonstrated greater stability than SICG between the two biocatalysts. There was a notable difference between two selected storage temperatures conditions, thus suggesting ideal storage temperature conditions for biocatalysts of this nature is 25 °C. When used as biocatalysts in chemical processes, the benefits listed above have also been shown to translate into lower operating costs (Chapman et al., 2018; Benítez-Mateos et al., 2021). As a result, the pharmaceutical, food, and beverage industries (Mamo and Assefa, 2018) have partially realized their lower energy requirements, waste mitigation, and simplified production routes (Zamel et al., 2019; Wei et al., 2021). However, more research is needed to show that biocatalysis is cost-effective in other industries like natural gas conversion and other bio-based production (Sheldon et al., 2021). In this regard enzyme immobilization will be instrumental in the realization of fully optimized biocatalysts, and the combination of technical expertise that will drive the scale-up of these economically competitive immobilized-biocatalytic processes for industrial applications.

We also investigated the thermal stability of the biocatalysts in comparison to the free enzyme because it is important for industrial laccase applications. As shown in Figure 6, an increase in temperature resulted in a decrease in free enzyme activity may be attributed to protein denaturation. (Budeli et al., 2020). also demonstrated that majority of isolated EDC-degrading bacteria were mesophiles, which they attribute to free laccase denaturation at high temperatures. Interestingly, the activities of both immobilized laccases remained unchanged at temperatures as high as 40 °C. For beads and SICG laccases, increasing the temperature to 100 °C resulted in only a 30% and a 66% loss of enzyme activity (Figure 6), respectively, representing a significant improvement in thermal stability of laccases. These biocatalysts are ideal for removing EDCs in combination with energy-intensive physical methods. Thermophiles have long been thought to be important in industry because they can be used in a variety of technological processes, either as whole organisms or as a source of thermostable enzymes that can catalyze specific reactions at high temperatures (Moreno et al., 2020). Free laccases became more stable under neutral pH, as evidenced by their ability to maintain maximum activities at pH 6 and 8 Figure 7. At acidic pH, free laccase forms showed complete exhaustion of activities. Environmental conditions such as pH has been shown to affect enzyme activity through changing the ionization status of functional groups within the enzyme (Sadr, 2014; Unuofin et al., 2019a). Immobilized enzyme forms were able to retain 40 and 50% enzymatic activity at pH 2, implying that immobilization improved laccase pH conditions. Immobilized laccase is thought to have a higher affinity for substrates than free laccase, possibly due to better orientation and availability of enzyme active sites (Moreno et al., 2020). However, due to activity loss, these two biocatalysts are most likely to have lower degradation efficiency under acidic conditions.

Immobilized enzyme should be stable and reusable for multiple cycles to make immobilization cost effective (Sigurdardóttir et al., 2018). After 28 cycles, laccase immobilized by beads retained 94% of its activity, while only 6% activity loss was observed at cycle 35 as depicted in Figure 7. SICG immobilised laccase, on the other hand, lost 12% of its activity after 21 cycles and remained constant until cycle 30. As a result of the findings, the immobilized laccases showed excellent recyclability. Immobilized enzymes are more resistant to heat, pH, storage, reuse, and other environmental factors than free enzymes. This could be due to the chemical bonds that connect the enzyme to the modified immobilization supports, which grant the enzyme configuration more stability and reduce the rate of enzyme leakage (Mohamad et al., 2015; Wahab et al., 2020). Both beads and SICG as immobilization supports retained 76% and 84% laccase, respectively. The use of SEM (Scanning electron microscope) and TEM (Transmission electron microscope) in biocatalysts design is critical, especially for improving binding affinity and preventing enzyme leakages (Liang et al., 2020). Future implementation prospects will need to take into account the structure-function relationship of the enzyme and the platform used in immobilization, as well as the optimized product yield at low implementation costs, and an integrated combinatorial strategy that combines experimental and computational approaches. Recyclability may also preclude the addition of freshly prepared enzymes on a regular basis.

Both biocatalysts were able to completely remove E2 as shown in Figure 9. Despite the fact that E1 and EE2 were not completely removed, both biocatalysts achieved a removal rate of over 95%. Bacterial species isolated from activated sludge have been linked to the transformation of E2 to E1 (Yu, Deeb and Chu, 2013; Zhang *et al*>, 2016). Throughout the study, the removal efficiency of beads immobilised laccase showed slightly higher removal percentages than SICG as depicted in Figure 9. The same trend was observed in degradation of EDCs using immobilisation supports without the enzymes. Overall, the results of this study showed that both immobilized laccase forms can be used to biodegrade EDC contaminated wastewater containing various estrogens. However, further research in larger scale continuous bioreactors is recommended to determine the technique’s efficacy and scalability. In general, beads immobilised biocatalysts were better in terms of stability, reusability, and efficacy; however, SICG-based biocatalysts are more likely to be adopted for real life applications due to their ease of synthesis and readily available precursors, impressive adaptability as well as lower production costs. This biocatalyst can also serve an additional function as a screen against bacterial pollution, due to the presence of the antibacterial properties from leaching silver (Budeli et al., 2021).

Despite the numerous benefits of biocatalysis, commercial enzyme catalysis is only used if it improves the process’s operational economics (Eş et al., 2015; Chapman et al., 2018). According to BCC research, the global enzyme market is expected to grow from $5.01 billion in 2016 to $6.32 billion in 2021, with market trends predicting a shift toward increased technical enzyme production, such as those used in the textile, paper, leather, and biodiesel industries, where excess waste generation results in environmental fines (Chapman et al., 2018; Basso and Serban, 2019; Ottone et al., 2020). Such market projections have been shown to be largely driven by process development in enzymatic biofuel production, biomass conversion, wastewater treatment etc, which present excellent opportunities for scaling-up of immobilized biocatalysts.

## Conclusion

Enzymatic activities of the two selected isolates revealed unequivocal involvement of bacterial laccases during estrogen degradation, suggesting that they could serve as alternative biological sources for estrogen removal in wastewater. However, the individual or interactive effects of enzymatic inoculum size (B) and Cu concentration (C) were particularly crucial for enhanced secretion. More than anything, the synthesized biocatalysts using beads and SICG indeed improve enzyme stability and efficiency as they were found to be more tolerant to temperature, pH, and other environmental perturbations. Correspondingly, they possessed improved storage ability and reusability, in addition to enhanced estrogen removal. Both biocatalysts were able to remove 100% of E2, as well as over 90% of E1 and EE2, within 24 h. Notwithstanding, they shared similar estrogen degradation patterns with free laccases, with E2 being the most removed, followed by E1 and lastly EE2. In comparison to clay based biocatalysts, beads biocatalysts possessed a higher potential for recyclability and were more resistant to environmental factors such as pH, storage, reuse, and temperature. The differences in terms of net estrogen removal was insignificant across all estrogens ranging between 95–100% and 94–100% for beads biocatalyst and clay biocatalyst, respectively. In view of the above credentials of the composite, the adoption of SIGC biocatalysts would be most feasible for real-life large-scale applications, due to its inexpensiveness and its additional function as an antibacterial composite. Therefore, future studies should be tailored to reduce the cutbacks it currently encounters[Bilal et al., 2018], [Kibambe et al., 2020].

## Data Availability

The raw data supporting the conclusions of this article will be made available by the authors, without undue reservation.
