# Supplementary material for: Novel bio-catalytic degradation of endocrine disrupting compounds in wastewater
Source: Front Bioeng Biotechnol. 2022 Oct 25;10:996566. doi: 10.3389/fbioe.2022.996566 (PMC9640757; doi:10.3389/fbioe.2022.996566)
Supplement: Supplementary file 1 [file DataSheet2.docx]

## Identification supplimentary

## 4.1. Identification and isolation of bacterial isolates

Distinct colonies were selected for purification by repeated streaking on nutrient agar until a single colony was obtained, resulting colonies were stored in stock media with glycerol at -20 °C for further identification using MALDI-TOF. As shown in **Table 1**, preliminary identification revealed the two selected bacterial isolates as *Lysinibacillus sp.* BP1 and *Lysinibacillus sp.* BP2 with a score value of 1.806 and 1.965, respectively.

**Table 1**: Identification of Isolates by MALDI-TOF

| Isolate | Organism (best match) | Score Value (SV) |
| --- | --- | --- |
| Slu 4 | *Lysinibacillus macrolides* | 1.965 |
| Slu 6 | *Lysinibacillus fusiformis* | 1.806 |

The analysis of the two assembled pairwise sequences alignment showed 100 % sequence identity and 82 % query coverage with *L. macrolides and 99 % sequence identity and 74 % query coverage for L. fusiformis* in similarity search using BLAST program**.** Based upon the highly conserved 16S rRNA sequences of selected isolates which matched with those of the other bacterial strains published in the NCBI database, close relatedness was seen with the genus *Lysinibacillus*; therefore, the isolated bacterial strains were named as *Lysinibacillus sp.* BP1 and *Lysinibacillus sp.* BP2.

Table 7.2: 16S rRNA sequence identification

| Isolate | Closest hit | ID (%) |
| --- | --- | --- |
| Slu-006 | *Lysinibacillus fusiformis* | 100 |
| Slu-004 | *Lysinibacillus macroides* | 99 |
