# Supplementary material for: Novel bio-catalytic degradation of endocrine disrupting compounds in wastewater
Source: Front Bioeng Biotechnol. 2022 Oct 25;10:996566. doi: 10.3389/fbioe.2022.996566 (PMC9640757; doi:10.3389/fbioe.2022.996566)
Supplement: Supplementary file 2 [file DataSheet1.docx]

**Annexture A**


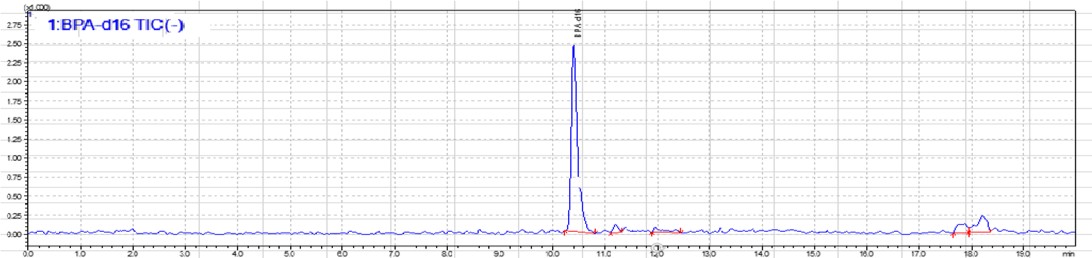
**Supplementary file:** Estrogen chromatograms


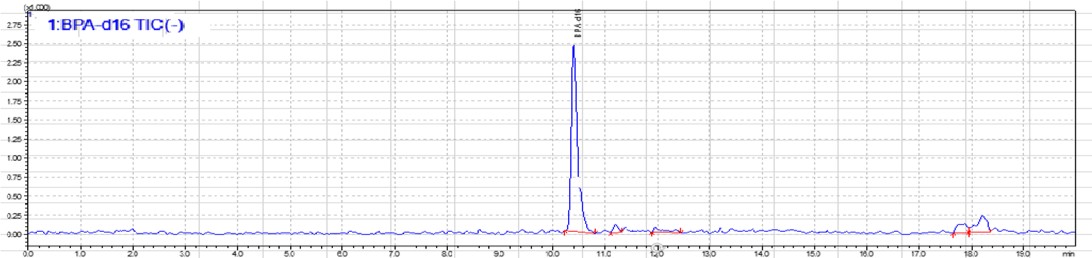


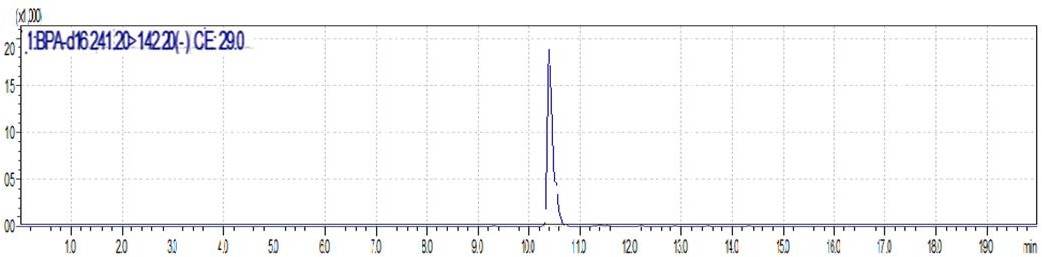


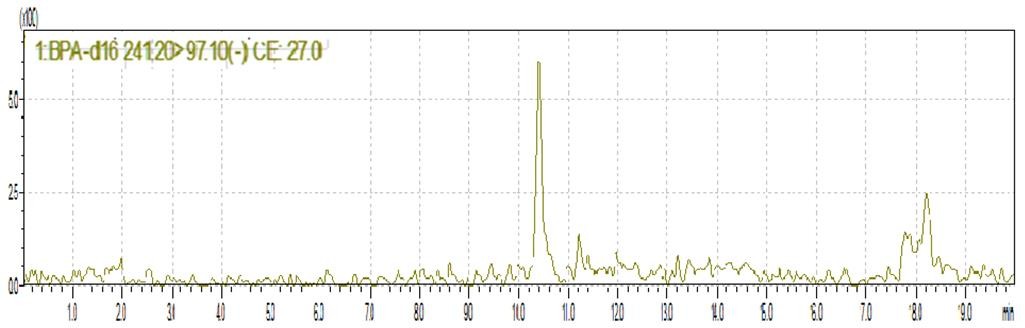


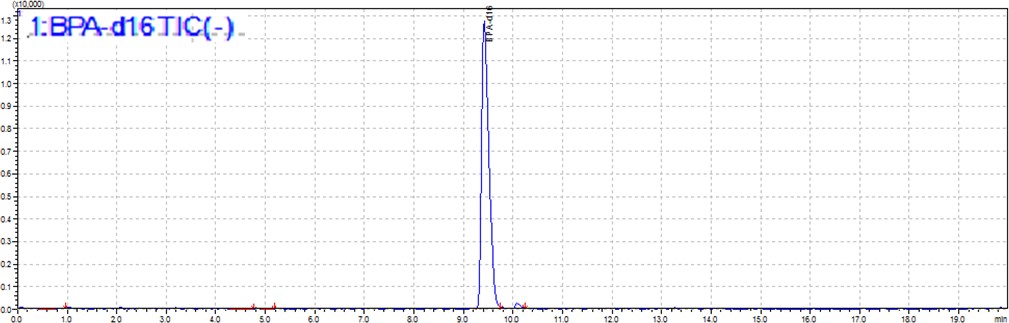


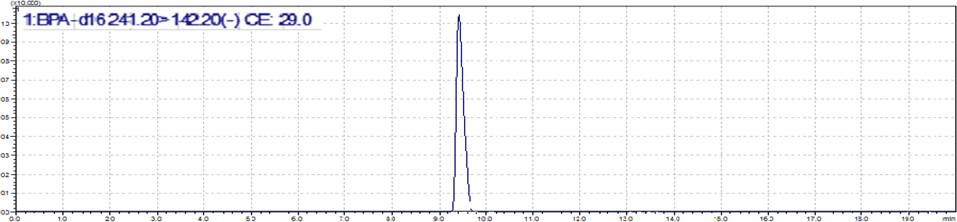


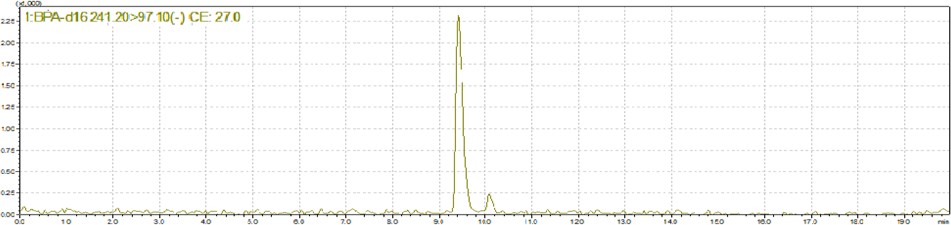


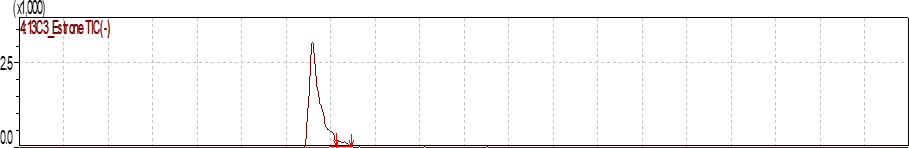


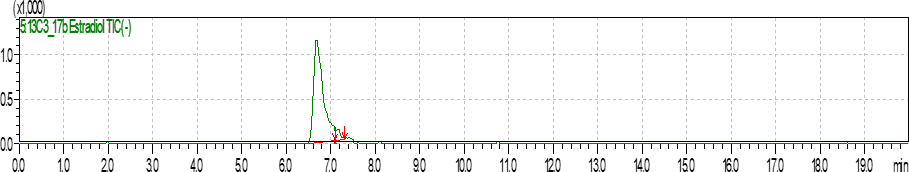


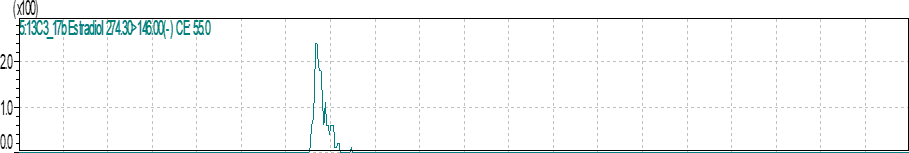


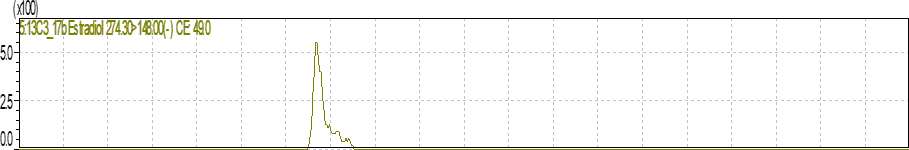


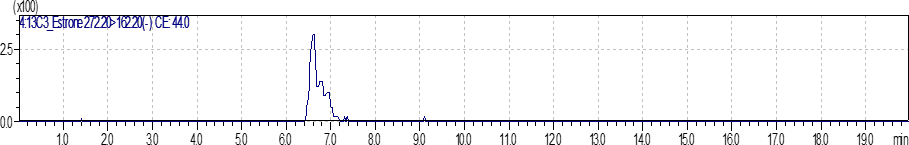


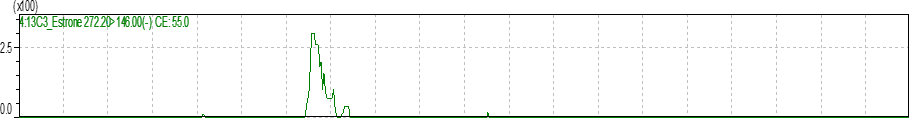


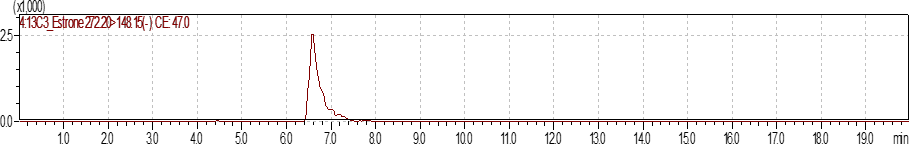


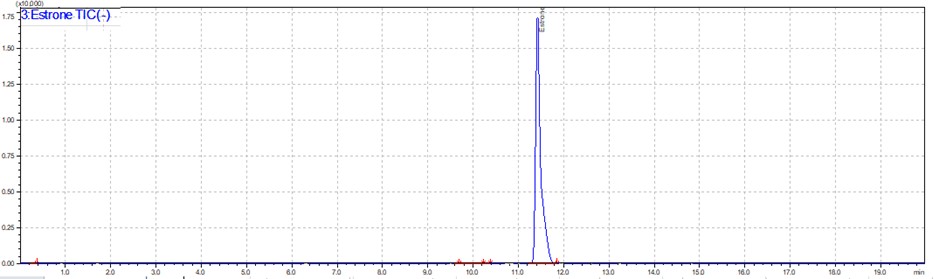


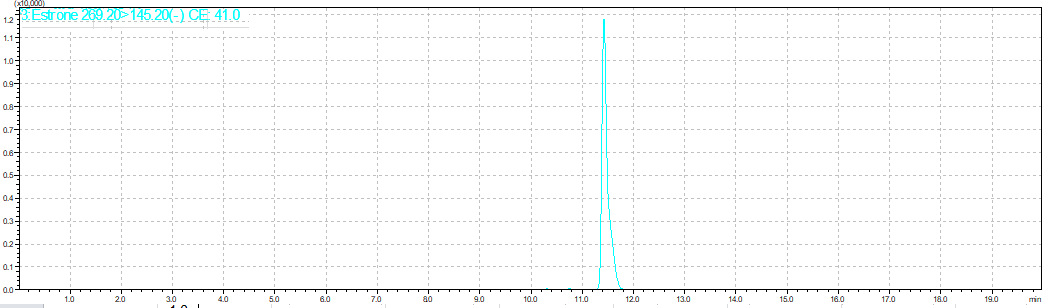


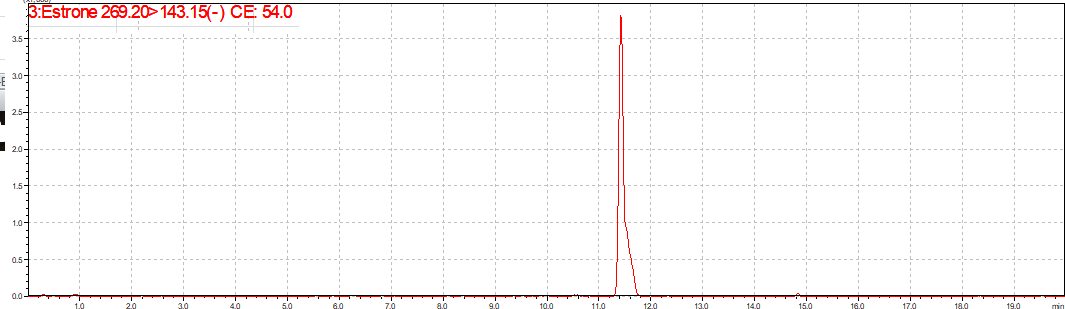


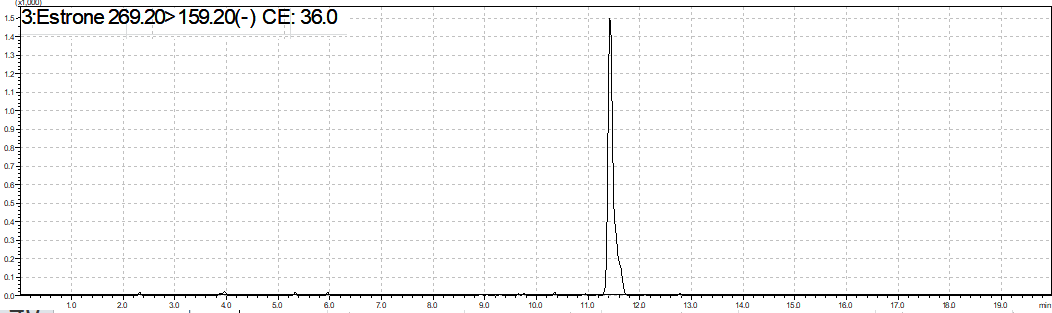


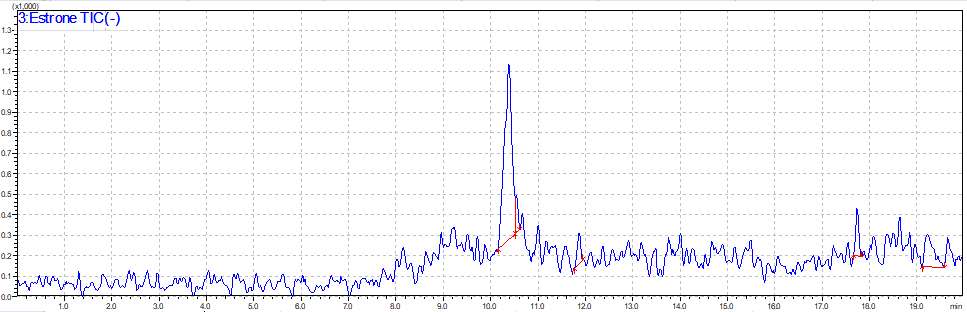


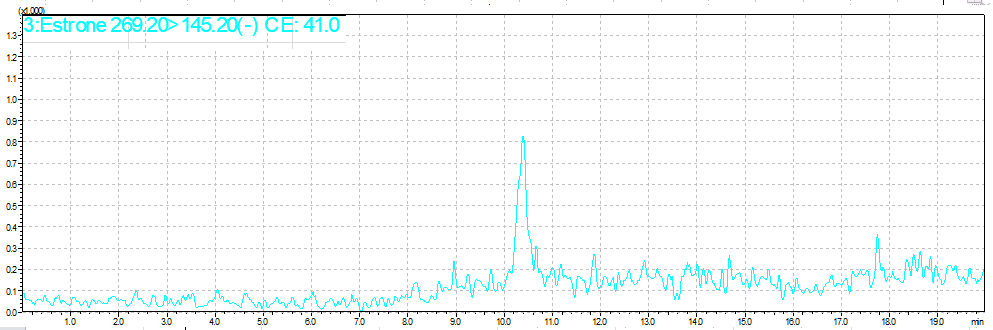


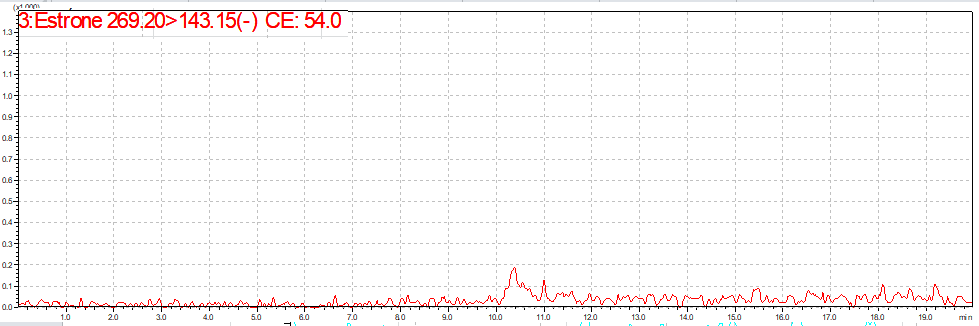


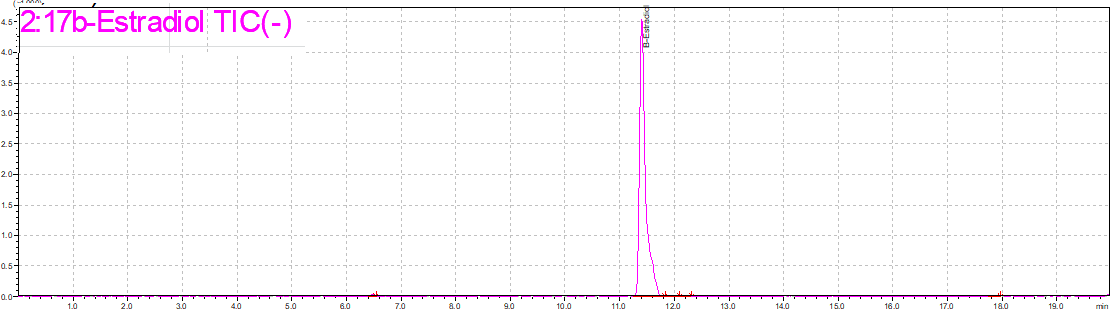


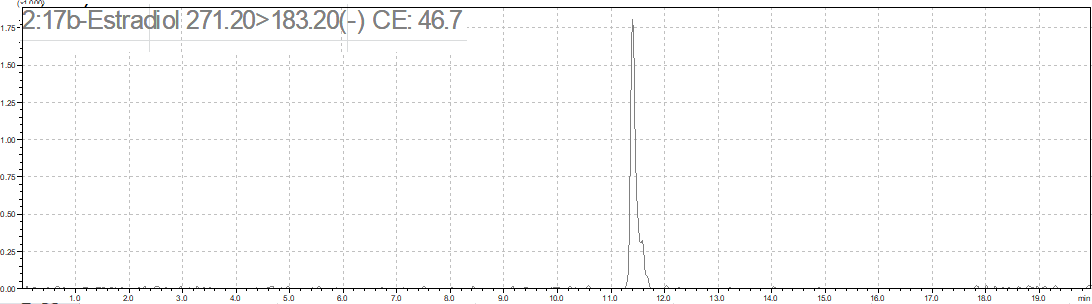


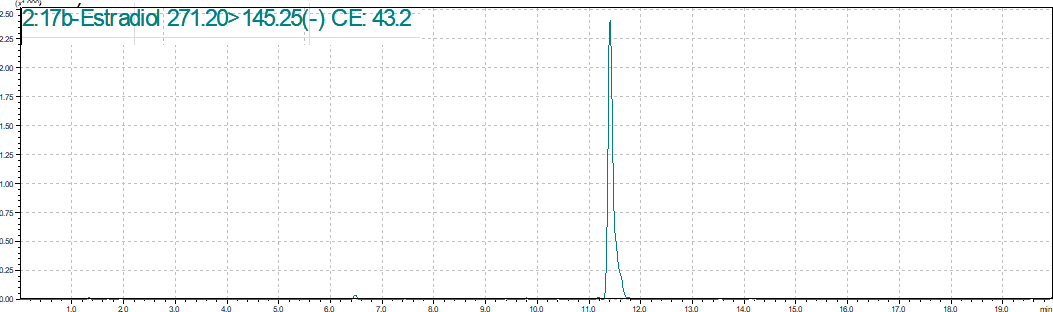


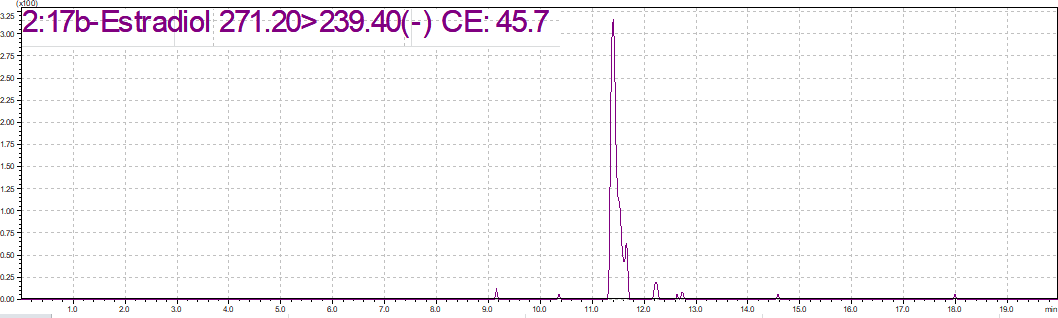


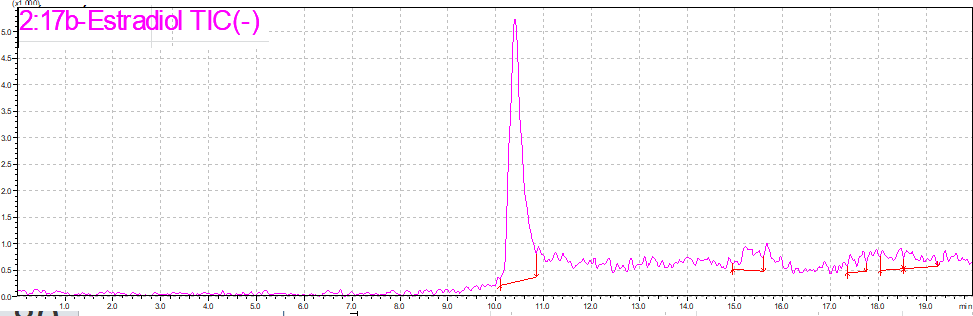


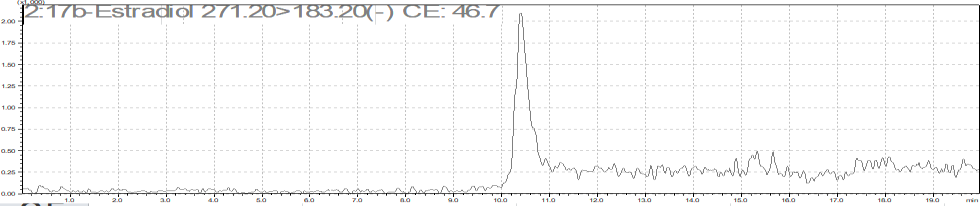


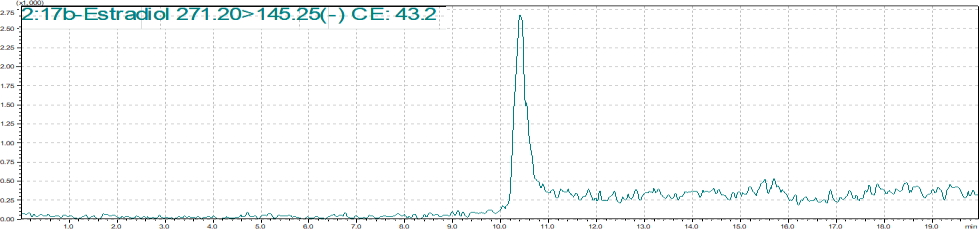


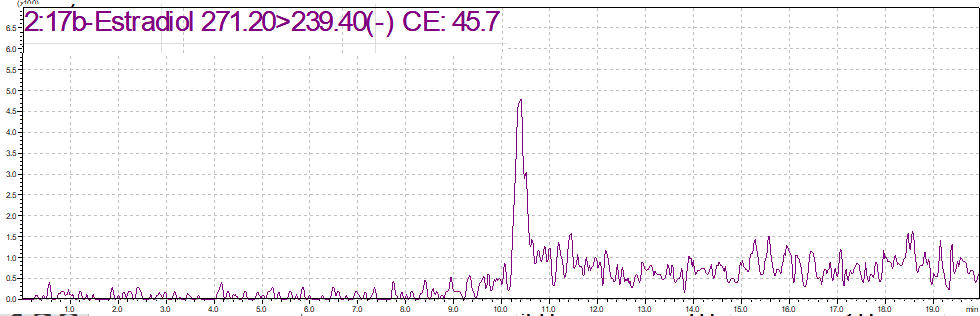


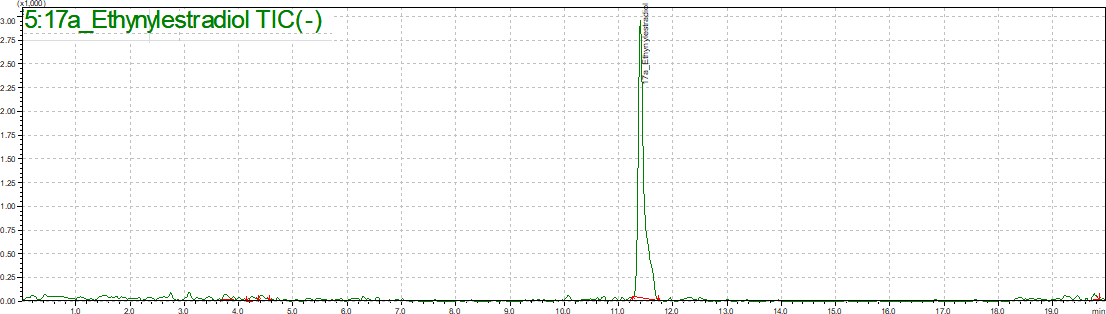


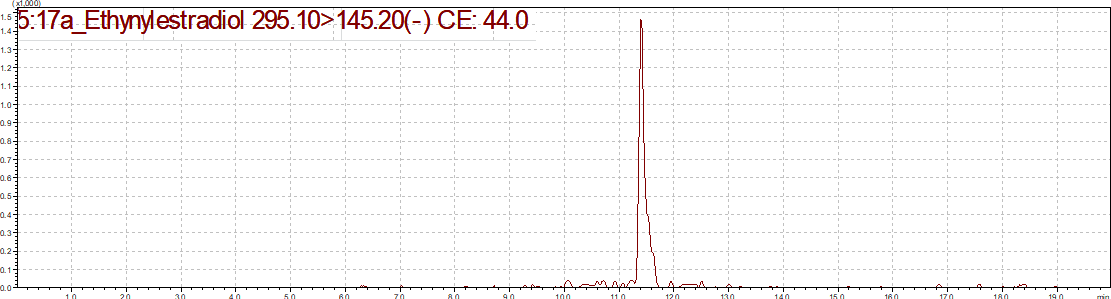


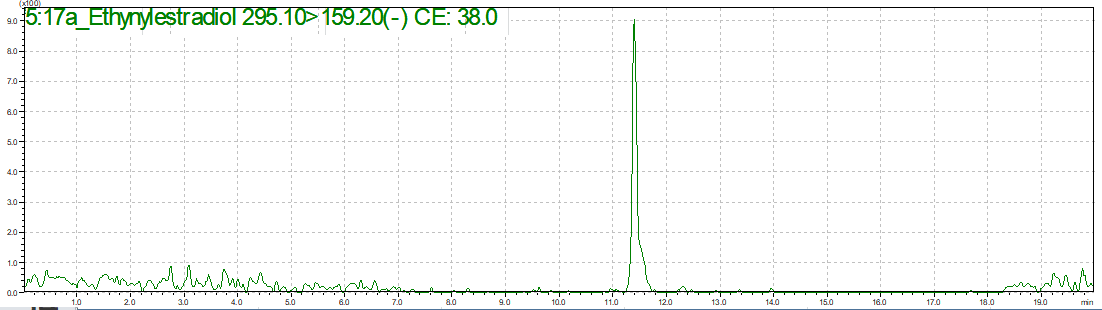


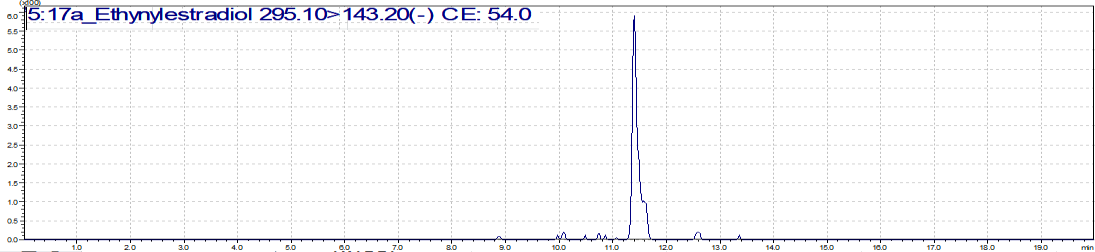


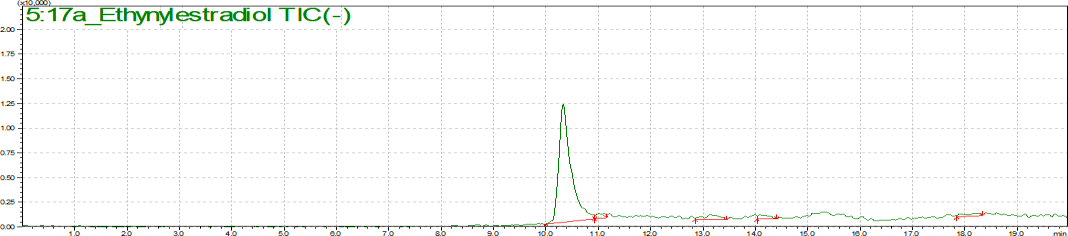


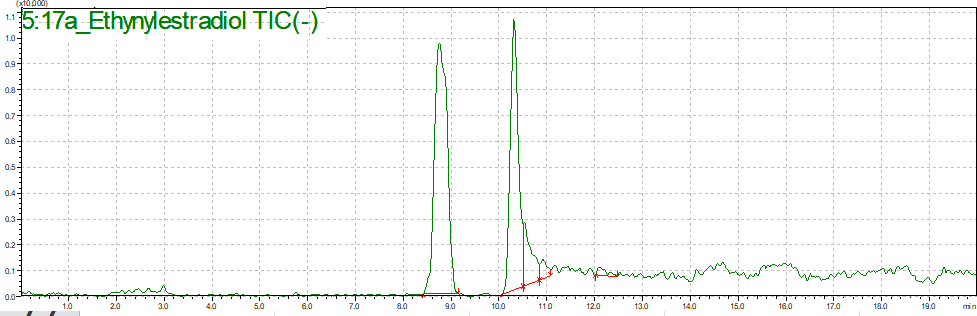


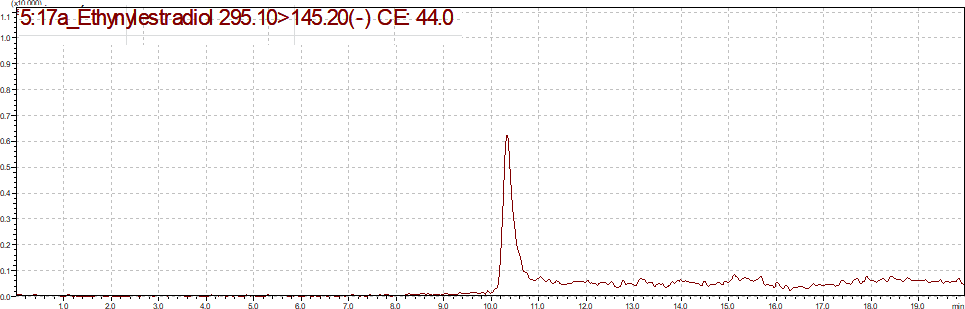


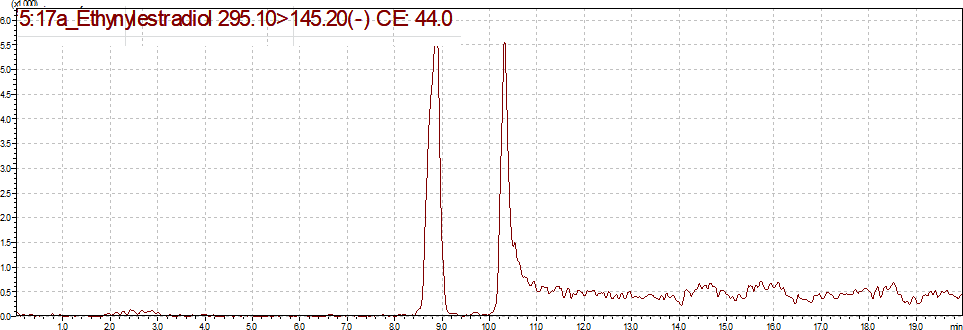


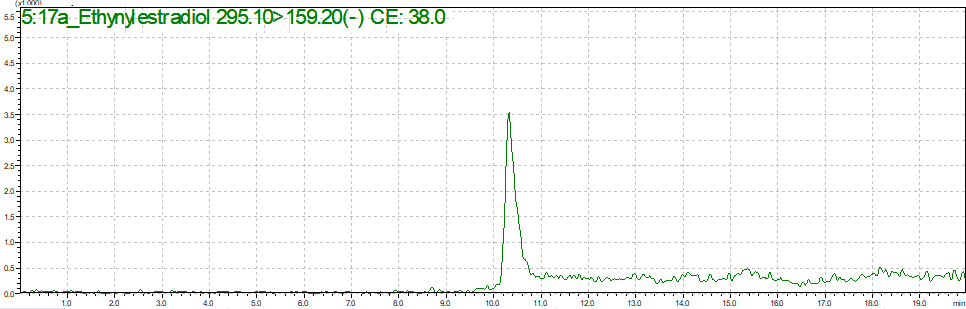


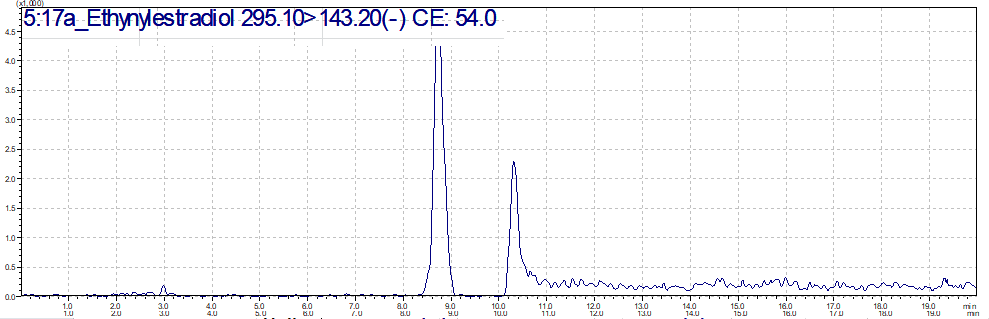

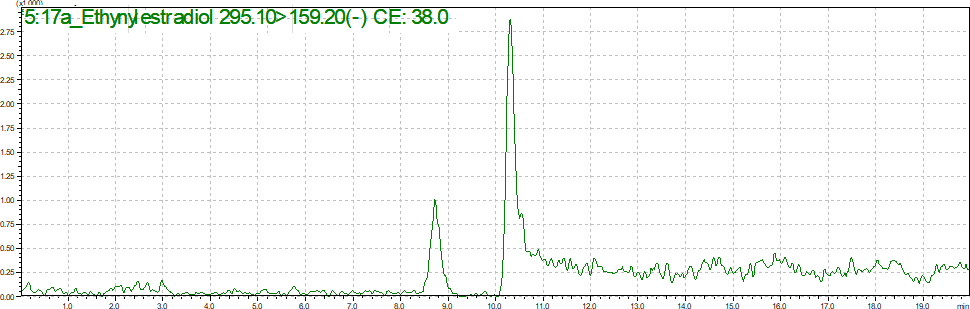


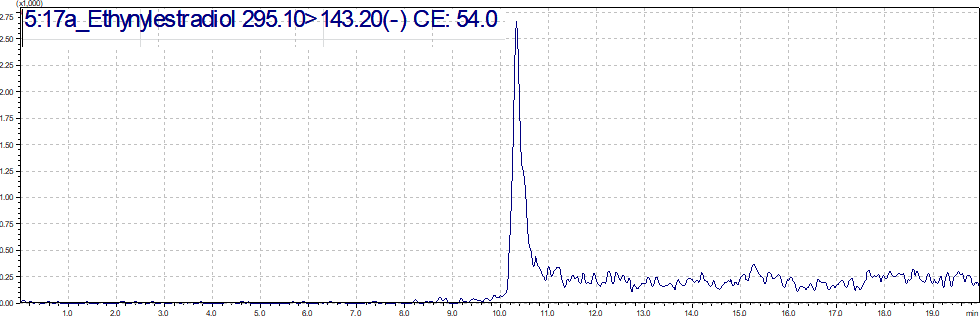


**Quality assurance**

To monitor for potential laboratory contamination, procedural blanks were extracted and analyzed alongside the extracted samples during the analysis. Methanol blanks were also used between samples to check for instrument contamination. As shown in Table 4.4, the calibration curve showed strong linearity with correlation coefficients >0.99 for all the target compounds. The limit of detection (LOD) and the limit of quantification (LOQ) from the calibration plots were defined as 3 and 10 times the signal-to-noise ratio, respectively. The LODs ranged from 0.38 ng/L to 1.30 ng/L and the LOQs ranged from 1.71 ng/L to 2.41 ng/L. Table 3.3 summarizes the analytical recovery of the target compounds as well as other method validation parameters.

## Table S2 : Method performance and validation parameters

| **Compound** | ***R*2** | **Mean recoveries (%)** | | **LOD (ng/L)** | **LOQ (ng/L)** |
| --- | --- | --- | --- | --- | --- |
|  |  | **10 ng/L** | **100 ng/L** |  |  |
| E1 | 0.9996 | 146 | 101 | 0.79 | 2.41 |
| E2 | 0.9993 | 70 | 113 | 0.38 | 1.71 |
| EE2 | 0.9992 | 75 | 90 | 0.60 | 1.82 |

# Table S3: Optimal MRM parameters for the studied estrogens

| **Compound** | **Precursor (m/z)** | **Product (m/z)** | **Cone voltage (V)** | **Collision energy (eV)** | **Q3 Pre Bias (V)** |
| --- | --- | --- | --- | --- | --- |
| E1 | 269.20 | **145.10**;  143.05 | 14 | 46 | **19.0**;  19.0 |
| E2 | 271.20 | **145.10**;  183.10 | 14 | 47 | **19.0**;  27.0 |
| EE2 | 295.30 | **145.05**;  159.05 | 11 | 47 | **17.0**;  25.0 |
